# Clusters of risk factors in metabolic syndrome and their influence on central blood pressure in a global study

**DOI:** 10.1038/s41598-022-18094-y

**Published:** 2022-08-24

**Authors:** Agne Laucyte-Cibulskiene, Chen-Huan Chen, John Cockroft, Pedro G. Cunha, Maryam Kavousi, Aleksandras Laucevicius, Maria Lorenza Muiesan, Ernst R. Rietzschel, Ligita Ryliskyte, Irina D. Strazhesko, Charalambos Vlachopoulos, Jorge Cotter, Ekatherina N. Dudinskaya, Nichola Gale, Fariba Ahmadizar, Francesco U. S. Mattace-Raso, Maggie Munnery, Pedro Oliveira, Anna Paini, Massimo Salvetti, Olga N. Tkacheva, Edward G. Lakatta, Peter M. Nilsson, Angelo Scuteri

**Affiliations:** 1 Department of Clinical Sciences, and Department of Nephrology, Lund University, Ruth Lundskogs gata 14, S-214 28 Malmö, Sweden; 2 Department of Medicine, School of Medicine, National Yang-Ming University, Taipei, Taiwan; 3 Cardiff Metropolitan University, Cardiff, UK; 4 Center for the Research and Treatment of Arterial Hypertension and Cardiovascular Risk, Internal Medicine Department, Guimarães, Portugal; 5 Life and Health Research Institute (ICVS/3B’s), Minho University, Braga, Portugal; 6 Department of Epidemiology, Erasmus University Medical Center, Rotterdam, The Netherlands; 7 Faculty of Medicine, Vilnius University, Vilnius University Hospital Santaros Klinikos, State Research Institute Centre of Innovative Medicine, Vilnius, Lithuania; 8 Department of Clinical and Experimental Sciences, University of Brescia, Brescia, Italy; 9 Ghent University Hospital, Ghent, Belgium; 10 The Stand-Alone Structural Unit of the Pirogov Russian National Research Medical University, the “Russian Clinical Research Center for Gerontology” of the Ministry of Healthcare of the Russian Federation, Moscow, Russia; 11 1st Department of Cardiology, National and Kapodistrian University of Athens, Athens, Greece; 12 School of Healthcare Sciences, College of Biomedical and Life Sciences, Cardiff, UK; 13 Department of Geriatrics, Erasmus MC University Hospital Rotterdam, Rotterdam, The Netherlands; 14 Departamento de Estudo de Populações, Instituto de Ciências Biomédicas Abel Salazar, Universidade Do Porto, Porto, Portugal; 15 Laboratory of Cardiovascular Sciences, National Institute on Aging – National Institutes of Health, Baltimore, USA; 16 Department of Clinical Sciences, Lund University, Malmö, Sweden; 17 Department of Medical Sciences and Public Health, University of Cagliari, Cagliari, Italy

**Keywords:** Cardiology, Risk factors

## Abstract

The effect of metabolic syndrome (MetS) and clusters of its components on central blood pressure (CBP) has not been well characterized. We aimed to describe the effect of MetS and clusters of its components on CBP in a large population and to identify whether this effect differs in men and women. We studied 15,609 volunteers (43% women) from 10 cohorts worldwide who participated in the Metabolic syndrome and Artery REsearch Consortium. MetS was defined according to the NCEP-ATP III criteria (*GHTBW*, glucose, high-density lipoprotein cholesterol, triglyceride, blood pressure, waist circumference). CBP was measured noninvasively and acquired from pulse wave analysis by applanation tonometry. MetS was associated with a 50% greater odds of having higher CSBP. After controlling for age, male sex, non HDL cholesterol, diabetes mellitus, and mean arterial pressure, only specific clusters of MetS components were associated with a higher CSBP; and some of them were significant in women but not in men. We identified “risky clusters” of MetS variables associated with high CSBP. Future studies are needed to confirm they identify subjects at high risk of accelerated arterial aging and, thus, need more intensive clinical management.

## Introduction

Brachial blood pressure measurement is the most widely used approach in managing hypertension in daily clinical practice. Many epidemiological and interventional studies showed an undeniable beneficial effect of lowering brachial blood pressure for cardiovascular (CV) and renal outcomes^1^. However, the accuracy of peripheral systolic and diastolic blood pressure in reflecting central blood pressure has been questioned since 2007^2^. This challenge has paralleled the increasing attention to large arteries. Indeed, recent research has focused on accelerated to healthy/supernormal vascular aging^3–5^ and the possible role of vascular geometry^6^; aortic stiffness as an independent predictor of cardiovascular morbidity^7^ and its association with the progression of cognitive impairment^8^ and multiple organ damage^9–11^; the definition of a “normality threshold” for clinical purposes^12^ and the role of arterial aging in response to treatment^13^; and the issue of central blood pressure in routine clinical practice^14^.

Whereas mean arterial pressure (MBP) and diastolic blood pressure (DBP) are relatively constant along the arterial tree, the height of the pressure pulse is amplified from the aorta toward peripheral arteries^15^. Therefore, brachial SBP is higher than central systolic blood pressure (CSBP). These differences decreases with advancing age and are affected by sex, body height, and cardiovascular risk factors (e.g., dyslipidemia, diabetes, and smoking)^16^.

As peripheral tissues to central (aortic) rather than brachial pressures, CBP has shown a stronger association with left ventricular hypertrophy, intima-media thickness, and pulse wave velocity^17^. And CBP was a stronger predictor of CV events than brachial BP^18–20^. Moreover, interventional studies showed that specific antihypertensive drug treatment differentially impacts on brachial and CSBP^21,22^.

Metabolic syndrome (MetS) is a complex construct encompassing several clusters of five components (low HDL cholesterol, increased fasting glucose, increased triglyceride, elevated waist circumference, and elevated peripheral blood pressure). The effects of Mets and the selected cluster of MetS components on large artery stiffness and thickness have been described in a previous report from the MARE Consortium^23^. However, the effect of MetS and clusters of MetS components on CBP has not yet been described in large populations.

The present cross-sectional, observational study aims to describe the association between MetS and selected clusters of its components on CBP in a large global population and identify possible sex differences in this association.

## Subjects and methods

### The MARE consortium

The original MARE (Metabolic Syndrome and Artery Research) Consortium aimed to identify diverse metabolic syndrome clusters and their association with vascular aging, gene-lifestyle interactions, and cardiovascular risk among ten cohort studies worldwide and to develop novel cardiovascular prevention methods based on lifestyle modification. The detailed methodology is published elsewhere^23^. The MARE Consortium is open to additional participating cohorts if data on the MetS components and arterial properties become available for the recruited subjects. The affiliates providing data for the present study are described in Appendix 1 and include subjects from Belgium, Portugal, Greece, Taiwan, Lithuania, Sweden, Russia, the Netherlands, and Italy. All participating countries provided approval for this study.

The ethical committee approved this international multicenter study of Ghent University Hospital and the University of Pennsylvania Institutional Review Board; the Committee of ethics of research with the medicine of the health area of Salamanca; the Ethics committee for the health of Guimaraes; the Ethics Committee of the Athens Medical School; Yu-li Veterans Hospital Ethics Committee; the Vilnius Regional Bioethics Committee of Clinical Research; the Ethical Committee at the Lund University; The ethics committee of the National Research Centre for Preventive Medicine in Moscow; The Medical Ethics Committee of Erasmus University; Bro Taf Local Research Ethics Committee in Cardiff; by the institutional Ethical Committee on human research of the University of Brescia (details provided in Appendix 1).

Each subject gave informed consent.

The MARE Consortium was performed in line with the principles of the Declaration of Helsinki and Title 45, U.S. Code of Federal Regulations, Part 46, Protection of Human Subjects, Revised November 13, 2001, effective December 13, 2001. All methods of this study were performed following the relevant guidelines and regulations stated in the Declaration of Helsinki.

### Definition of the metabolic syndrome

MetS was defined according to The Third Report of the National Cholesterol Education Program Expert Panel on Detection, Evaluation, and Treatment of High Blood Cholesterol in Adults (NCEP ATP III)^24^ criteria, where the metabolic syndrome is diagnosed if three or more of the following five components are present:

1. Elevated fasting glucose (G) (≥ 110 mg/dl) or the presence of drug treatment for increased glucose;
2. Low HDL cholesterol (H) (< 40 mg/dl in men and < 50 mg/dl in women) or the presence of a specific treatment for lipid abnormalities;
3. High triglycerides (T) (≥ 150 mg/dl) or the presence of a particular treatment for lipid abnormalities;
4. Elevated blood pressure (systolic or diastolic, ≥ 130 or ≥ 85 mmHg) (B) or presence of antihypertensive treatment;
5. Abdominal obesity (W) with a waist circumference of more than 102 cm in men and more than 88 cm in women.

Since at least three components define metabolic syndrome, the study subjects could have had 16 different MetS component combinations.

### Brachial and central blood pressure measurements

Brachial blood pressure (BP) was measured according to European Society of Hypertension recommendations^25^. Pulse pressure (PP) was determined as systolic BP minus diastolic BP in mmHg. Mean arterial pressure (MBP) calculated as diastolic BP + 1/3 (PP).

After resting for 10 min in the supine position, central blood pressure was measured noninvasively and acquired from pulse wave analysis by applanation tonometry (SphygmoCor, AtCor Medical Pty Ltd, Sydney, Australia) at the femoral artery or common carotid artery. The heart rate was monitored using three-lead electrocardiography. All measurements were performed by a trained operator three times in a row; the goal operator index was considered greater than 80%. Only measurements that fulfilled these requirements were analyzed. Elevated central systolic BP was defined as higher than or equal to 140 mmHg.

The gold standard method for CBP is invasive measurement. According to the previous reports^26^, the applanation tonometry derived CBP overestimates the invasively measured CBP by 0.3 ± 1.0 mmHg.

### Statistical analysis

All analyses were performed using the SAS package for Windows (9.1 Version Cary, NC, US). ANOVA followed by the Bonferroni test was adopted to compare means among subgroups of subjects. Least square means (± standard error, SEM) were calculated with ANCOVA analysis to compare CSBP, CPP, and pulse pressure amplification (PPA) values and to compare CSBP values across clusters of MetS components after controlling for covariates (age, sex, non HDL cholesterol (nonHDL-C) levels, MBP, presence of diabetes mellitus). To test for possible age- or sex-specific differences in CSBP and CPP across MetS clusters, interaction terms for sex, age, and MetS clusters were alternatively introduced into separate models.

Multivariable logistic regression models were constructed to identify potential clusters of MetS components associated with high CSBP (= > 140 mmHg).

A two-sided p-value < 0.05 indicated statistical significance.

## Results

The characteristics of the 15,609 participants (43% women) from cohorts participating in the MARE consortium are illustrated in Table 1. CPP, but not CSBP, was significantly greater in women than in men (51 ± 18 vs. 49 ± 17 mmHg, p < 0.001).

**Table 1:** Characteristics of the study cohorts from the MARE Consortium.

Variable	N	Value
Age, years	15,609	59 (14)
Sex (men)	15,609	57% (8897)
MI	9245	5.8% (536)
Stroke	8767	3.4% (298)
Hypertension	12,168	55% (6692)
Diabetes mellitus	15,035	12% (1804)
Smoking	15,490	50% (7745)
Antihypertensive treatment, yes	11,314	24% (2715)
Andiabetic treatment, yes	7931	7.9% (626)
Lipid lowering drugs, yes	11,565	17% (1966)
Brachial SBP, mmHg	15,609	136 (20)
Brachial DBP, mmHg	15,609	79 (11)
Brachial PP, mmHg	15,609	57 (16)
Mean arterial blood pressure, mmHg	15,609	98 (13)
Central SBP	15,609	130 (21)
Central PP, mmHg	15,609	50 (17)
Heart rate, bpm	13,196	70 (12)
BMI, kg/m2	13,995	27.4 (4.8)
Waist circumference, cm	15,503	93 (13)
Total cholesterol mg/dL/mmol/L	15,303	217 (47)/5.6 (1.2)
High density cholesterol, mg/dL/mmol/L	15,236	57 (19)/1.5 (0.5)
Triglycerides, mg/dL/mmol/L	15,205	121 (108)/1.4 (1.2)
Glucose, mg/dL/mmol/L	14,078	101 (25)/5.6 (1.4)
Creatinine, mg/dL/mkmol/L	14,692	0.88 (0.24)/78 (21)
cfPWV, m/s	15,609	9.9 (3.3)
CCA IMT, mm	10,957	817 (227)

Data are presented as the average (SD) for continuous measures and percentage (n) for categorical measures.

Abbreviations: BMI, body mass index; MI, myocardial infarction; SBP, systolic blood pressure; DBP, diastolic blood pressure; PP, pulse pressure; CCA IMT, common carotid artery intima-media thickness; cfPWV, carotid-femoral pulse wave velocity.

### Effects of specific clusters of MetS components on CSBP

**Figure 1:**
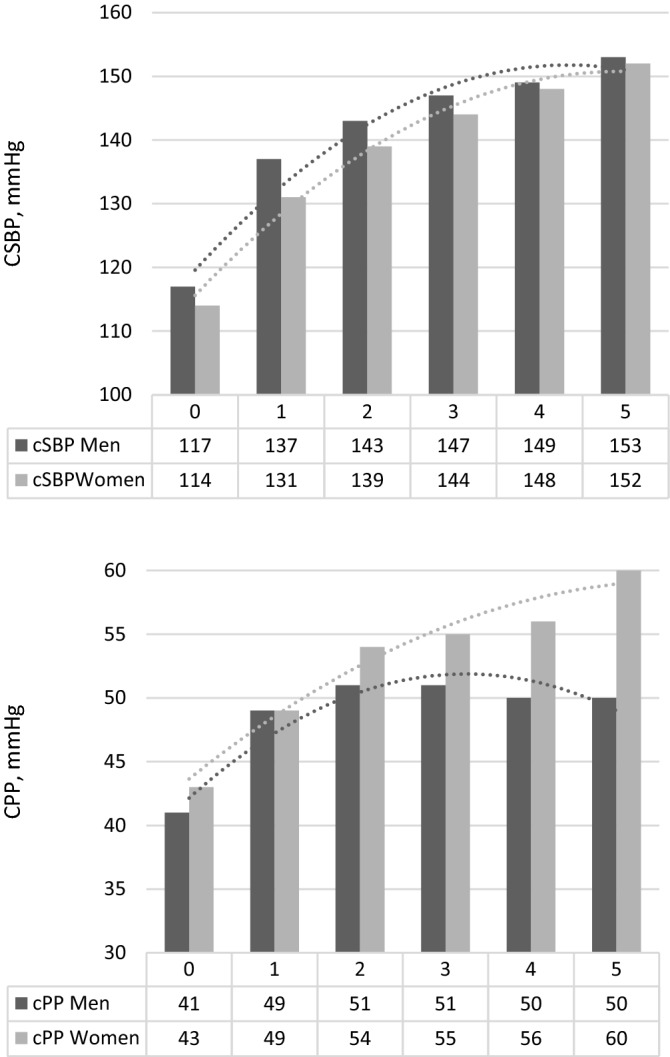
Central systolic blood pressure and pulse pressure values according to the number of altered MetS components. Men—black bars, women—gray bars. Numbers from 0 to 5 indicate the numbers of MetS components. Significant sex-specific differences in CSBP (p < 0.001) and in CPP (p < 0.001). Three components or more are considered typical of MetS.

Both CSBP and CPP levels progressively increased with the number of altered MetS components (Fig. 1).

In the model including age, sex, brachial SBP and DBP, and use of antihypertensive medications, MetS was associated with a 50% greater odds of having high CSB (OR 1.50; 95% CI: 1.38–1.63, p < 0.001).

Then we tried to identify specific clusters of MetS components associated with higher CSBP, as previously illustrated for large artery stiffness and thickness. As expected, a cluster of MetS components with elevated brachial BP had higher CSBP and CPP levels (data not shown) than those MetS clusters without elevated BP levels. Therefore, to account for differences in brachial BP levels, glucose, and lipid levels according to the cluster of MetS components, multivariable logistic regression models were constructed, including non HDL-C, MBP, and presence of diabetes mellitus—together with age and sex—as covariates; and controlling for age, male sex, nonHDL-C, MBP, and presence of diabetes mellitus.

**Figure 2:**
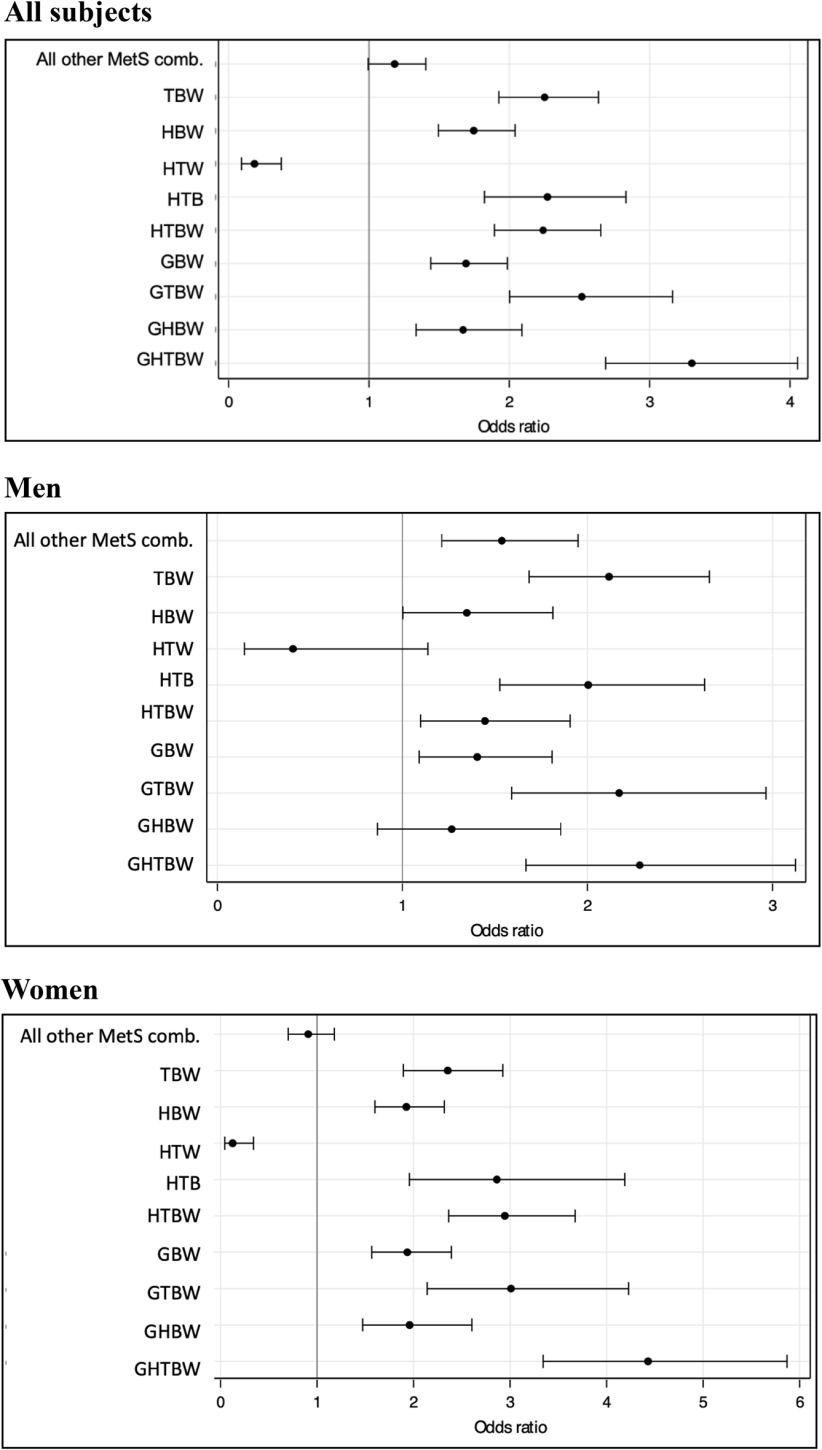
Clusters of MetS components as determinants of high CSBP—controlling for age, diabetes mellitus, nonHDL-C, and mean blood pressure. Odds ratio (OR) with 95% confidence interval for specific MetS clusters of components in the whole population (upper panel), in men (central panel), and women (lower panel). We evaluated all the possible combinations of MetS components, but only specific clusters are described here. Abbreviations: All other MetS comb., all different combinations of metabolic syndrome components not depicted in the picture; MetS, metabolic syndrome; W, abdominal obesity; H, low HDL cholesterol; B, high blood pressure; T, triglycerides; G, glucose; MAP, mean arterial pressure. Not significant clusters in men: *HTW, HBW, GHBW*; in women: *HTW*; in the whole population: *HTW*.

Except for *low HDL-C-hypertriglyceridemia-abdominal obesity* (*HTW*), the other combinations of MetS components were accompanied by a 1.3 to a 4.0 fold greater odds of presenting high CSBP (Fig. 2). Of note, in addition to HTW, HBW and GHBW MetS clusters were not associated with greater CSBP levels in men but not in women (Fig. 2 bottom panels).

Secondary analyses, run after excluding participants using antihypertensive medications, showed that the cluster of MetS components TBW (OR 1.52, 05% CI 1.05–2.20), HTB (OR 1.85, 95% CI 1.15 -2.97), and GHTBW (OR 3.40, 95% CI 1.99–5,81) were associated with higher CSBP after controlling for age, sex, diabetes, non HDL cholesterol, and MBP levels.

## Discussion

The present cross-sectional, observational study showed that MetS was associated with greater odds of having higher CSBP, independent of age, sex, and brachial blood pressure levels. However, not all the clusters of MetS components defining “the metabolic syndrome” were associated with high CSBP, and sex-differences were observed in the specific MetS clusters associated with high CSBP.

Large arteries are heterogeneous, and their functional and structural properties are poorly correlated with each other^27,28^. Measures of large artery structure and function as markers of vascular aging have emerged as independent predictors of CV morbidity and related disability^29^.

Notably, specific clusters of MetS components—namely HBW, TBW, and GBW—have been constantly associated with greater odds not only of high CSBP but also of stiffer^23^ and thicker^29^ arteries.

Given the cross sectional-nature of the MARE Consortium, including large population studies, we can only speculate about potential pathophysiological mechanisms underlying our findings.

The first interpretation—identifying a bias rather than a finding—may suggest that MetS clusters, including the “elevated (brachial) blood pressure” (B) component, carried greater odds of having high CSBP simply because of the high correlation between brachial and central SBP levels.

Though we cannot rule out this interpretation, it does not seem to represent the most accurate explanation. When subjects receiving antihypertensive treatment were excluded, not all the MetS clusters, including the “elevated (brachial) blood pressure” (B) component, were associated with significantly greater odds of having high CSBP.

Additionally, in men, specific MetS clusters, including elevated brachial blood pressure (HBW and GHBW), were not associated with greater odds of having high CSBP.

Furthermore, a significant association between HTW and high CSBP had been expected, but it has not been observed in the present study. In fact, visceral adiposity has been associated with a fivefold higher risk of hypertension^30^. Recently, the adipose tissue has emerged as an endocrine organ, secreting adipokines (adiponectin, pectin, etc.) with a systemic impact on the cardiac and vascular system. Lower adiponectin levels were observed in the presence of higher ambulatory 24-h blood pressure in the *Porto Alegre* cohort^31^. Lower adiponectin levels have been associated with stiffer arteries independently of MetS components in the SardiNIA Study^32^ and hypertensive subjects with MetS^33^. Adipose tissue also expresses mineralocorticoid receptors, modulating vascular remodeling, development of glucose tolerance, and obesity^34^.

A relevant unanswered question remains whether these clusters recognize a common altered pathway of pathophysiological relevance—with or without a genetic basis—remains speculative to date. However, these MetS clusters likely identify subjects with accelerated arterial aging, at greater risk of CV mortality and disability, and, thus, need a more intensive management.

Despite ongoing research collaborations and activities in gender-related science, further research is needed to analyze the sex-specific interplay between MetS and central hemodynamics. Two decades ago^35^, different large artery properties showed elevated CBP in men younger than 40 years compared to women. The Bogalusa Heart Study^36^ observed a more pronounced discrepancy between peripheral and central blood pressure in women than in men. The SardiNIA Study reported a stronger association of visceral obesity with arterial stiffness in women than in men^37^. Gender differences have also been reported for genetic markers of visceral adiposity^38^. We also report a steeper CSBP slope with age in women than in men, with a turning point at 50 years of age, after which the difference disappears. Our study observed higher CPP in women not dependent on MetS components such as elevated brachial blood pressure and abdominal obesity.

Yet, the sex-specific differences in central hemodynamics in the context of MetS still need clarification.

One limitation of this study is represented by its cross-sectional design. Additionally, no information on antihypertensive drug classes was universally coded and available for participants from all MARE Consortium cohorts.

This multicenter, multiethnic observational study confirms that the greater odds of having high CSBP associated with the presence of MetS hides a constellation of phenotypes that are not equally risky of arterial aging, whether indexed as central BP, large artery stiffness, or thickness.

Identification of “risky clusters” in the whole population and specific to sex may contribute to a more personalized management of CV risk and lead to the identification of novel pathways accelerating arterial aging.

Further studies are needed to elucidate the sex-specific interplay between MetS components and the pathophysiology of vascular aging.

## Supplementary Information

Supplementary Information 1.

Supplementary Information 2.
