# Supplementary material for: Clusters of risk factors in metabolic syndrome and their influence on central blood pressure in a global study
Source: Sci Rep. 2022 Aug 24;12:14409. doi: 10.1038/s41598-022-18094-y (PMC9402529; doi:10.1038/s41598-022-18094-y)
Supplement: Supplementary file 1 — Supplementary Information 1. [file 41598_2022_18094_MOESM1_ESM.doc]

**Appendix 1.** Brief description of the participating cohorts

**Asklepios Study**

The Asklepios Study is an extensively phenotypic population-representative random sample of Belgian volunteers free from clinically overt CV disease at baseline. An in-depth description of the Asklepios study protocol has been published (1). Their data were collected between 2002 and 2004.

This study was approved by the ethical committee of Ghent University Hospital and the University of Pennsylvania Institutional Review Board, and all subjects gave written informed consent.

**Guimarães/Vizela study**

It is a population-based study conducted in two adjacent cities (Guimarães and Vizela) of the region of Alto Minho (Portugal), characterized by one of the highest incidences of stroke in Europe. The cohort included 3038 randomly selected subjects, men and women, from age 18-96 (55% women). Each subject underwent a detailed medical history, blood pressure and anthropometric measurements, a 12-lead resting EKG, blood and urine analysis, measurements of arterial stiffness and central blood pressure, and (for subjects older than 50 years) cognitive testing (2).

The study has been approved by Committee of ethics of research with medicines of the health area of Salamanca” in 14/12/2018 (cod. 2018-11-136) and the “Ethics committee for health of Guimaraes” (Portugal) in 15/10/2019 (ref: 67/2019). All subjects gave written informed consent.

**HYGEIA (Hippocrattes hYpertension, aGEIng and Arterial function)**

It is a cohort of 1225 naïve hypertensive subjects recruited in Athens, Greece, of broad age range (19-84 years) (HYGEIA-HTN) and a cohort of 466subjects from the community-dwelling population of the Greek island of Ikaria (HYGEIA-AGE) (3).

The HYGEIA protocol was approved by the Ethics Committee of the Athens Medical School, and participants signed written informed consent form

**Kinmen Aging Study**

In 1992 to 1993, 2230 Chinese residents in Pu-Li town and Kinmen County, Taiwan, were invited to participate in a comprehensive cardiovascular survey, including medical history and physical examination, arterial tonometry and ultrasonography, echocardiography, 24-hour ABPM, and biochemical examinations in the fasting state (4).

All participants gave informed consent, and the study was approved by the institutional review board at the Johns Hopkins University.

Ethics approval was obtained from the Yu-Li Veterans Hospital Ethics Committee, and all subjects gave written informed consent.

**Lithuanian High Cardiovascular Risk Study (LitHiR)**

LitHiR is a prospective study conducted in Lithuania. Caucasian subjects with metabolic syndrome (originally defined according to the updated NCEP definition) with no previous CV disease underwent full medical examination, which included a 12-lead resting ECG, exercise tolerance test, echocardiogram, blood tests for metabolic and lipid profile, and measurements of arterial structure and function. Their data were collected between 2007 and 2012.

The protocol was approved by the Vilnius Regional Bioethics Commintee of Clinical Research (08-11-2011 nº 158200-11-417-117), and all subjects gave written informed consent.

**Malmö Diet and Cancer – Cardiovascular Cohort**

The Malmö Diet and Cancer (MDC-CC) study is a population-based, prospective epidemiologic cohort of 28 449 persons enrolled between 1991 and 1996 aged 45 to 69 years. From this cohort, 6103 persons were randomly selected to participate in the MDC cardiovascular cohort, which was designed to investigate the epidemiology of carotid artery disease (5).

The MDC-CC protocol was approved by the Ethical Committee at the Lund University in 1990 at baseline screening with reference number LU 51-90, and all subjects gave written informed consent.

**Moscow Study**

Moscow Arterial Aging Study is a prospective study conducted in the National Research Centre for Preventive Medicine in Moscow, Russia, from May 2012 to December 2013. We excluded subjects with previous history of drug medication for diabetes, hypertension or hyperlipidaemia, a history of cardiovascular diseases as well as a cancer. 303 subjects (104 males and 199 females) were recruited. The subjects ranged between 23 and 91 years of age, with a mean age of 51.8 ±13.3 years. Of the study group, 76 subjects had mild or moderate hypertension, 50 subjects had T2DM, and 33 subjects were diagnosed with impaired glucose tolerance. None of the patients with T2DM had known microvascular or macrovascular complications. Patients completed examination which included measurements of arterial stiffness, intima-media thickness, endothelium-dependent vasodilation, metabolic, hormonal status, telomere biology, gut microbiota. IMT data were available for 287 subjects (6).

Informed consent was signed by all participants. Data privacy was ensured by using anonymized identifiers. Protocol of the study was approved by the local ethics committee, meeting #8, 29 November 2011.

**Rotterdam Study**

The Rotterdam Study (RS) is a prospective population-based cohort study comprising 7,983 subjects (78% response rate) aged 55 years or older. Baseline data (RS-I) were collected between 1990 and 1993. In 1999, inhabitants who turned 55 years of age or moved into the study district since the start of the study were invited to participate in an extension of the RS (RS-II) of whom 3,011 participated (67% response rate). The rationale and design of the RS have been described in detail elsewhere (7).

The Medical Ethics Committee of Erasmus University approved the study, and all subjects gave written informed consent.

**SMART Study**

The SMART Study was designed to investigate the effect of individual risk factors in isolation and clustered on arterial properties and CV events. The cardiovascular risk was also assessed using the ESC Heart score tool. Subjects undergo periodical follow-up to look at outcome data.

The SMART study was approved by Bro Taf Local Research Ethics Committee in Cardiff, UK, and all subjects gave written informed consent.

# **Vobarno Study**

The Vobarno Study was conducted in Vobarno, a small town in Northern Italy. Subjects were selected from the electoral rolls of the town (response rate 66%) (8). The data concerning 385 subjects, mean age 57±10 years (35-65 years) were available for the present analysis.

The Vobarno study was approved by the institutional Ethical Committee on human research of the University of Brescia in 1992, and all subjects gave written informed consent.

**References**

1. Rietzschel ER, De Buyzere ML, Bekaert S, Segers P, De Bacquer D, Cooman L, et al.; Asklepios Investigators. Rationale, design, methods and baseline characteristics of the Asklepios Study. Eur J Cardiovasc Prev Rehabil. 2007 Apr;14(2):179-91. .
2. Cunha PG, Cotter J, Oliveira P, Vila I, Boutouyrie P, Laurent S, el al. Pulse wave velocity distribution in a cohort study: from arterial stiffness to early vascular aging. J Hypertens. 2015 Jul;33(7):1438-45.
3. Vlachopoulos C, Xaplanteris P, Vyssoulis G, Bratsas A, Baou K, Tzamou V, et al. Association of serum uric acid level with aortic stiffness and arterial wave reflections in newly diagnosed, never-treated hypertension. Am J Hypertens. 2011 Jan;24(1):33-9.
4. Chen CH, Ting CT, Lin SJ, Hsu TL, Chou P, Kuo HS, et al. Relation between diurnal variation of blood pressure and left ventricular mass in a Chinese population. Am J Cardiol. 1995 Jun 15;75(17):1239-43.
5. Laucevičius A, Rinkūnienė E, Petrulionienė Ž, Ryliškytė L, Jucevičienė A, Puronaitė R, et al. Trends in cardiovascular risk factor prevalence among Lithuanian middle-aged adults between 2009 and 2018. Atherosclerosis. 2020 Apr;299:9-14.
6. Hedblad B, Nilsson P, Janzon L, Berglund G. Relation between insulin resistance and carotid intima-media thickness and stenosis in non-diabetic subjects. Results from a cross-sectional study in Malmö, Sweden. Diabet Med. 2000 Apr;17(4):299-307.
7. Strazhesko I, Tkacheva O, Boytsov S, Akasheva D, Dudinskaya E, Vygodin V, et al. Association of Insulin Resistance, Arterial Stiffness and Telomere Length in Adults Free of Cardiovascular Diseases. PLoS One. 2015 Aug 26;10(8):e0136676.
8. Hofman A, Breteler MM, van Duijn CM, Janssen HL, Krestin GP, Kuipers EJ, et al, Vingerling JR, Witteman JC. The Rotterdam Study: 2010 objectives and design update. Eur J Epidemiol. 2009;24(9):553-72.
9. Muiesan ML, Salvetti M, Zulli R, Pasini GF, Bettoni G, Monteduro C, et al. Structural association between the carotid artery and the left ventricle in a general population in Northern Italy: the Vobarno study. J Hypertens. 1998 Dec;16(12 Pt 1):1805-12.
