# Supplementary material for: Clusters of risk factors in metabolic syndrome and their influence on central blood pressure in a global study
Source: Sci Rep. 2022 Aug 24;12:14409. doi: 10.1038/s41598-022-18094-y (PMC9402529; doi:10.1038/s41598-022-18094-y)
Supplement: Supplementary file 2 — Supplementary Information 2. [file 41598_2022_18094_MOESM2_ESM.docx]

**Supplemental Table 1.** Determinants of cPP stratified for sex following adjustment for age, systolic and diastolic blood pressure, antihypertensive treatment and nonHDL-C.

|  | **Women** | | | **Men** | | |
| --- | --- | --- | --- | --- | --- | --- |
|  | **ß** | **SE** | ***P*** | **ß** | **SE** | ***P*** |
| G | -1.65 | 0.39 | <0.001 | -1.96 | 0.29 | <0.001 |
| H | -2.06 | 0.38 | <0.001 | -2.61 | 0.27 | <0.001 |
| T | 3.28 | 0.41 | <0.001 | 3.09 | 0.30 | <0.001 |
| B | -0.58 | 0.41 | 0.165 | -0.76 | 0.30 | 0.011 |
| W | -0.49 | 0.36 | 0.277 | -1.52 | 0.23 | <0.001 |
| MetS | 0.15 | 0.56 | 0.789 | 1.41 | 0.39 | 0.0003 |

Multiple linear regression analysis with cPP as dependent variable.

Abbreviations: MetS, metabolic syndrome; W, abdominal obesity; H, low HDL cholesterol; B, high blood pressure; T, elevated triglycerides; G, elevated glucose.
